# Exploration of ecological restoration of saline-alkali land based on NbS——Study on the salt resistance and desalination performance of three cash crops

**DOI:** 10.1371/journal.pone.0275828

**Published:** 2022-10-10

**Authors:** Jing Li, Youjiang Diao, Lihua Jiang, Qiuyue He, Fangzhi Wang, Wenfeng Hao

**Affiliations:** 1 School of Resource and Environmental Engineering, Shandong Agriculture and Engineering University, Jinan, China; 2 Binzhou Jingyang Biological Fertilizer Co., Ltd., Binzhou, China; KGUT: Graduate University of Advanced Technology, ISLAMIC REPUBLIC OF IRAN

## Abstract

Soil salinization is one of the current global environmental problems. Current research on crops in saline-alkali land focuses on salt tolerance, but less on its ecological benefits. However, plants and the environment can interact and influence each other, which is the theory used to carry out Nature-based Solutions (NbS). Therefore, the research on crop plants with both ecological and economic benefits is novel and valuable work. Then three widely planted cash crops (*Solanum melongena*, *Momordica charantia*, *Capsicum annuum*) were selected for salt stress treatment (NaCl, 150mmol/L), some physiological indicators (chlorophyll, soluble protein, Proline (Pro), malondialdehyde (MDA)) of plant and the soil properties (electrical conductivity, pH, the soil salt content) were measured. The results showed that the salinity content of the three plant cultivation soils was significantly different (P<0.05) after the salt stress; all three crops had some desalination capacity, but *Capsicum annuum* had the strongest salt resistance and desalination capacity.

## Introduction

Soil salinization is one of the current global environmental problems. The decrease of crop yield and cultivated land resulting from soil salinization have seriously affected the global food security and restricted the sustainable development of agriculture. The global saline-alkali soil area is 1.1×10^9^ hm^2^, and China has about 3.69×10^7^ hm^2^ [1]. Meanwhile, global climate change further aggravates soil salinization [2]. Therefore, the study of highly salt-tolerant plants has always been a research highlights.

If the plant can grow normally in saline-alkali land and can absorb salt from soil at the same time, it is more worthy of planting and promotion. This is the mechanism of biological improvement of saline-alkali land. A study on the utilization of different saline-alkali land in the Yellow River Delta found that the salt content of farmland was the lowest, which was significantly better than that of forest land and saline-alkali wasteland [3]. Besides, the soil nutrient status and enzyme activity of saline-alkali farmland were also the best [3]. Therefore, it is worth considering the use of crops with high salinity resistance in the biological improvement of saline-alkali land.

From the perspective of economic development, the cultivation of salt-tolerant crops in saline-alkali land can improve land utilization and increase farmers’ income. This promoted the development of agriculture.

From the perspective of ecological restoration of saline-alkali land, the planting of salt-tolerant plants can increase the ground cover and reduce the evaporation of soil surface water, thereby reducing the accumulation of salt in the soil. In addition, plants can absorb salt and fix them in the plant during the growth process, so as to achieve the effect of desalination. The improvement of saline-alkali land by the power of nature, thereby promoting the stability of the ecosystem, is just the philosophy of Nature-based Solutions (NbS). Nature-based Solutions are actions to protect, sustainably manage, and restore natural and modified ecosystems that address societal challenges effectively and adaptively, simultaneously providing human well-being and biodiversity benefits. This concept guides us to rely on nature power to protect and restore the ecosystem, resulting in healthy ecosystems and a stable/biodiverse future.

Therefore, combining the two perspectives of economic benefits and ecological benefits, it is more urgent to develop crops with salt tolerant and desalination ability to grow in salty conditions. Many scholars have studied the salt tolerant characteristics of plants in saline-alkali land [4–19]. Among them, varieties with higher salt tolerance are found in crops, such as sorghum, wheat, cotton, soybean [14–19], etc. Research on saline-alkali land in China even dates back to the 1950s [20]. However, there are relatively few studies based on the above two characteristics about crops (tolerant and desalination ability).

Considering this issue, three widely planted cash crops are selected for study to understand their salt tolerance and desalination capacity in this paper. Several typical resistance indexes of the plants after the stress were determined, like chlorophyll, soluble protein, Proline (Pro), malondialdehyde (MDA) [4–19, 21, 22]. And some soil-related factors, like soil salt content, soil conductivity and pH value are determined, including before and after salt stress. Then the desalination capacity of the three cash crops could be able to preliminary understand by comparing the soil salt content of different crops under the same treatment. It is expected that this research will open up some new research ideas, which will not only help to solve the aggravation of global environmental problems caused by the continuous expansion of saline-alkali land, but also improve the saline-alkali soil while ensuring farmers’ income.

## Materials and methods

### Plant material

The seedling samples of three kinds of cash crops, *Solanum melongena*, *Momordica charantia* and *Capsicum annuum*, were provided by Zibo Seed Service Station of Shandong. The sowing time was in mid-February, the seedlings were moved into pots (14*11*13cm) to be treated after 50 days of raising. The soil used for the experiment was taken from the 0-10cm topsoil of the idle farmland in the campus of Shandong Agriculture and Engineering University. Then the same weight of soil was placed in each pot and 5 seedlings in each pot. The single salt solution (NaCl, 150 mmol/L) was watered once a day (25 ml per pot) for a total of 10 days.

### Measurement method

Seedling leaves were randomly sampled every 2 days (4–5 replicates) to determine the chlorophyll, soluble protein, Pro and MDA content. Chlorophyll content was measured by SPAD-502 plus (Konica Minolta); soluble protein content was determined by the Biuret method [23], the Pro content was determined by the Ninhydrin method [23], MDA content was determined by the Thiobarbituric Acid method [23].

The soil characteristics before and after salt stress treatment were measured. The soil electrical conductivity and pH value are determined by extraction method, and the soil salt content is determined by gravimetric method [24]. Besides, the soil salinization grade is divided according to the measured values, the classification standard of saline-alkali land is shown in Table 1 [25]. The lower the salt content in the soil, the lower the grade, which is more suitable for plant growth.

**Table 1:** Salinization grade standard.

Grade	Ⅰ	Ⅱ	Ⅲ	Ⅳ	Ⅴ
**soil salt content(g/kg)**	≤0.97	0.97~1.86	1.86~3.91	3.91~6.27	>6.27

### Statistical analysis

Significant differences between species and among multiple means in the concentration of each indicator within species were tested using One -Way ANOVA, Duncan test (P<0.05) (SPSS v. 16.0).

## Results

In this experiment, the control group was watered with water, the treated group was irrigated with a single salt solution (NaCl, 150 mmol/L). Each physiological index of cash crops were measured every 2 days.

### Chlorophyll

**Fig 1:**
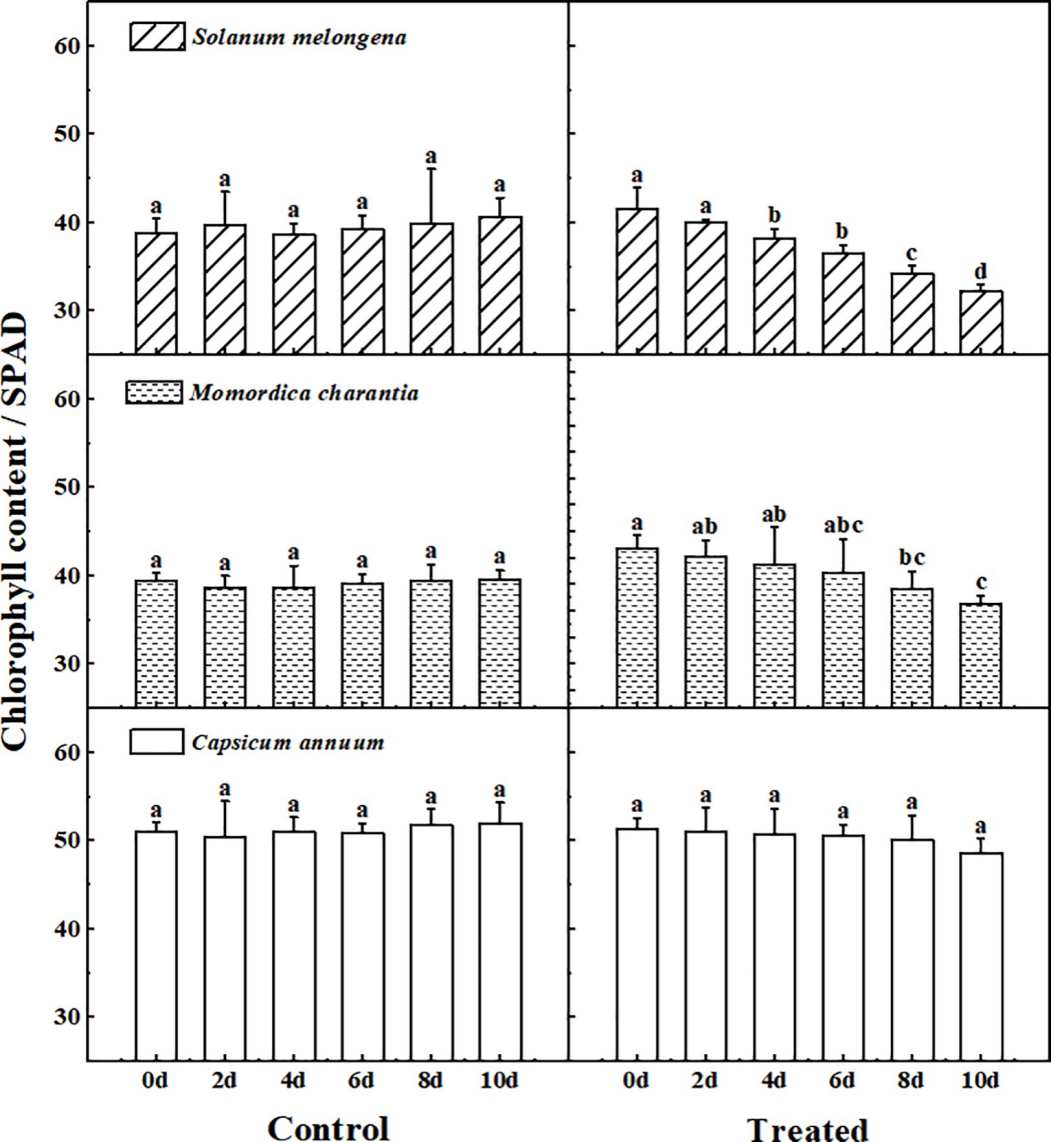
The response of chlorophyll to normal watering (Control) and salt water irrigation (Treated) in *Solanum melongena*, *Momordica charantia*, *Capsicum annuum*. The single salt solution (NaCl, 150 mmol/L) was watered once a day (25 ml per pot) for a total of 10 days, seedling leaves were randomly sampled every 2 days to determine the chlorophyll. Error bars represent standard errors of Chlorophyll with 4–5 replicates measurements. The same letter in each picture represents no significant difference (Duncan test, P < 0.05, n = 4–5).

Fig 1 shows the chlorophyll determination results of the control and treated group of three cash crops. The data showed that the interspecific differences of three crops in chlorophyll content were significant (P<0.05), and the chlorophyll content of *Capsicum annuum* was significantly higher than that of the other two cash crops. The chlorophyll content of *Solanum melongena* and *Momordica charantia* showed a gradually decreasing trend after salt stress, and the differences within the group were obvious (P<0.05). However, there was no obvious change in *Capsicum annuum* after stress, there was no significant difference from the control group.

### Soluble protein

**Fig 2:**
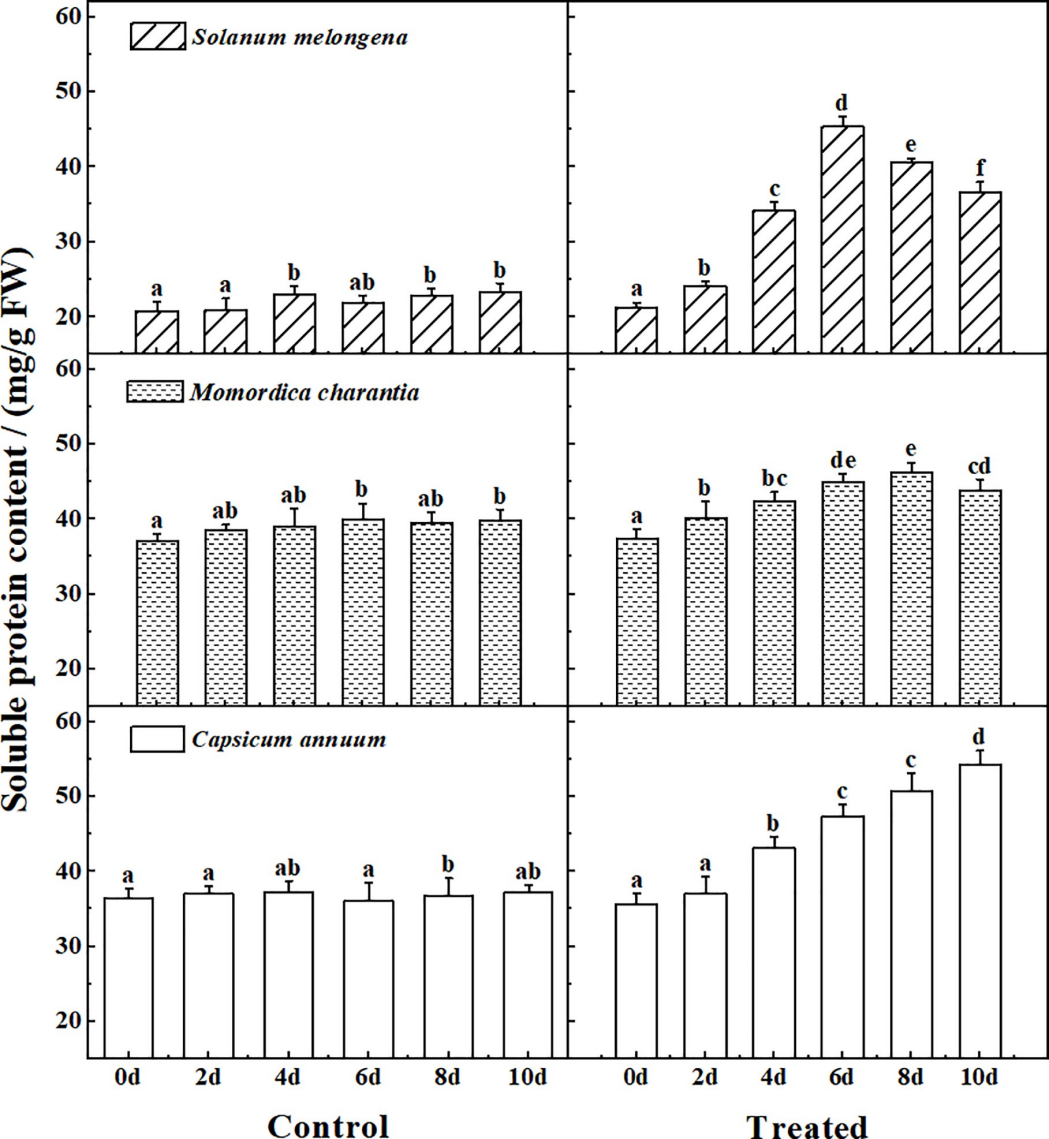
The response of soluble protein to normal watering (Control) and salt water irrigation (Treated) in *Solanum melongena*, *Momordica charantia*, *Capsicum annuum*. The single salt solution (NaCl, 150 mmol/L) was watered once a day (25 ml per pot) for a total of 10 days, seedling leaves were randomly sampled every 2 days to determine the soluble protein content. Error bars represent standard errors of soluble protein content with 4–5 replicates measurements. The same letter in each picture represents no significant difference (Duncan test, P < 0.05, n = 4–5).

Fig 2 shows the soluble protein determination results of the control and treated group of three cash crops. From the data of the control group, it can be seen that *Momordica charantia* and *Capsicum annuum* have high content, which are significantly higher than that of *Solanum melongena* (P<0.05). During salt stress treatment, the soluble protein content of *Solanum melongena* and *Momordica charantia* show a trend of first increasing and then decreasing, but the rate of change in *Solanum melongena* is more pronounced. With the prolongation of stress time, the soluble protein content of *Capsicum annuum* gradually increased, and the difference within the group was obvious (P<0.05).

### Proline

**Fig 3:**
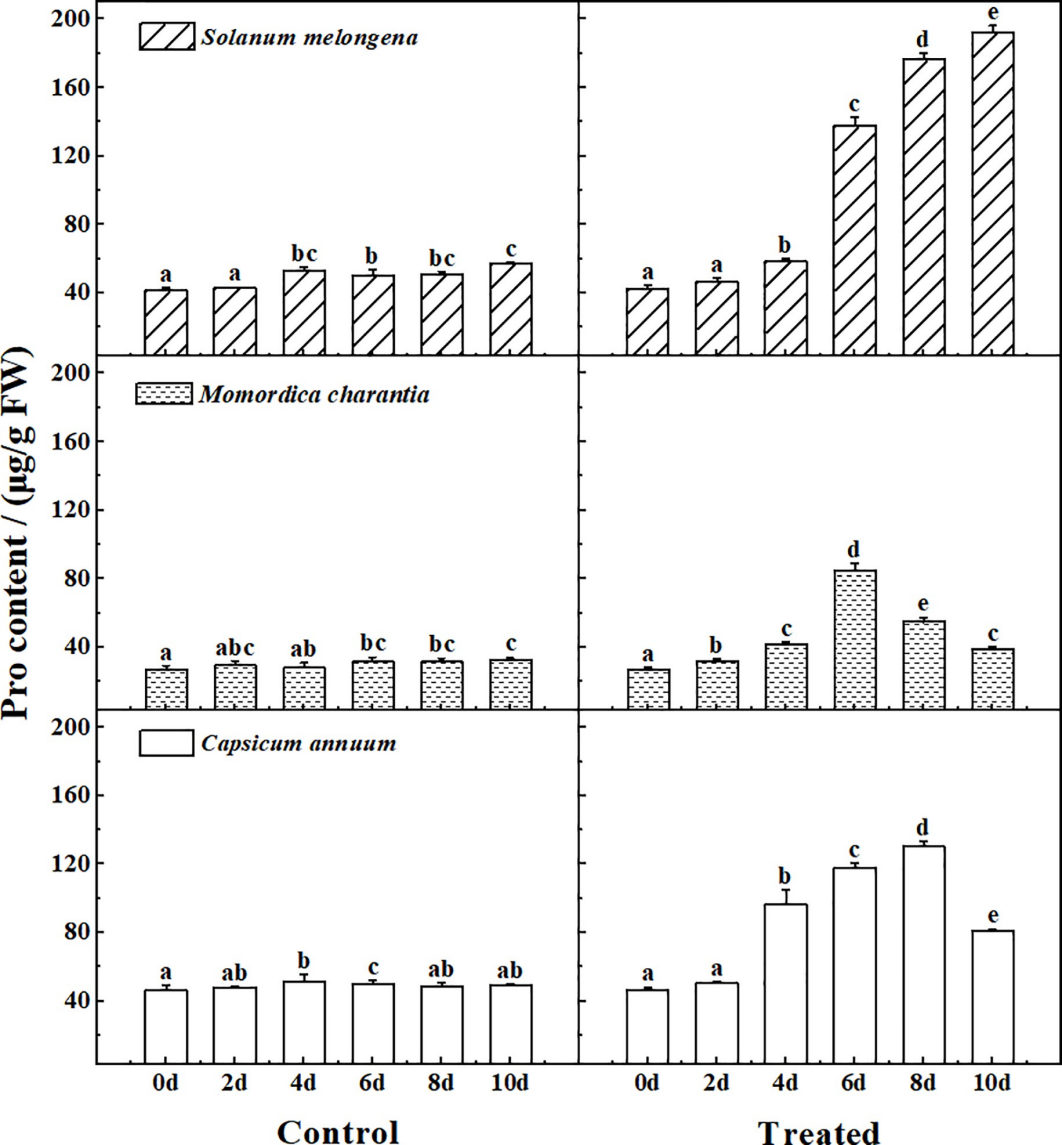
The response of proline (Pro) to normal watering (Control) and salt water irrigation (Treated) in *Solanum melongena*, *Momordica charantia*, *Capsicum annuum*. The single salt solution (NaCl, 150 mmol/L) was watered once a day (25 ml per pot) for a total of 10 days, seedling leaves were randomly sampled every 2 days to determine Pro. Error bars represent standard errors of Pro with 4–5 replicates measurements. The same letter in each picture represents no significant difference (Duncan test, P < 0.05, n = 4–5).

Fig 3 shows the Pro determination results of the control and treated group of three cash crops. From the data of the control group, it can be seen that the Pro content of *Momordica charantia* is significantly lower than that of the other two crops. Significant changes (P<0.05) in Pro content were observed in the three crops after salt treatment. After saline watering, the Pro content of the three plants showed an upward trend in the short-term stress, but decreased in *Momordica charantia* and *Capsicum annuum* at the later stage of stress. The Pro content of *Solanum melongena* increased more than 3 times after salt stress.

### Malondialdehyde

**Fig 4:**
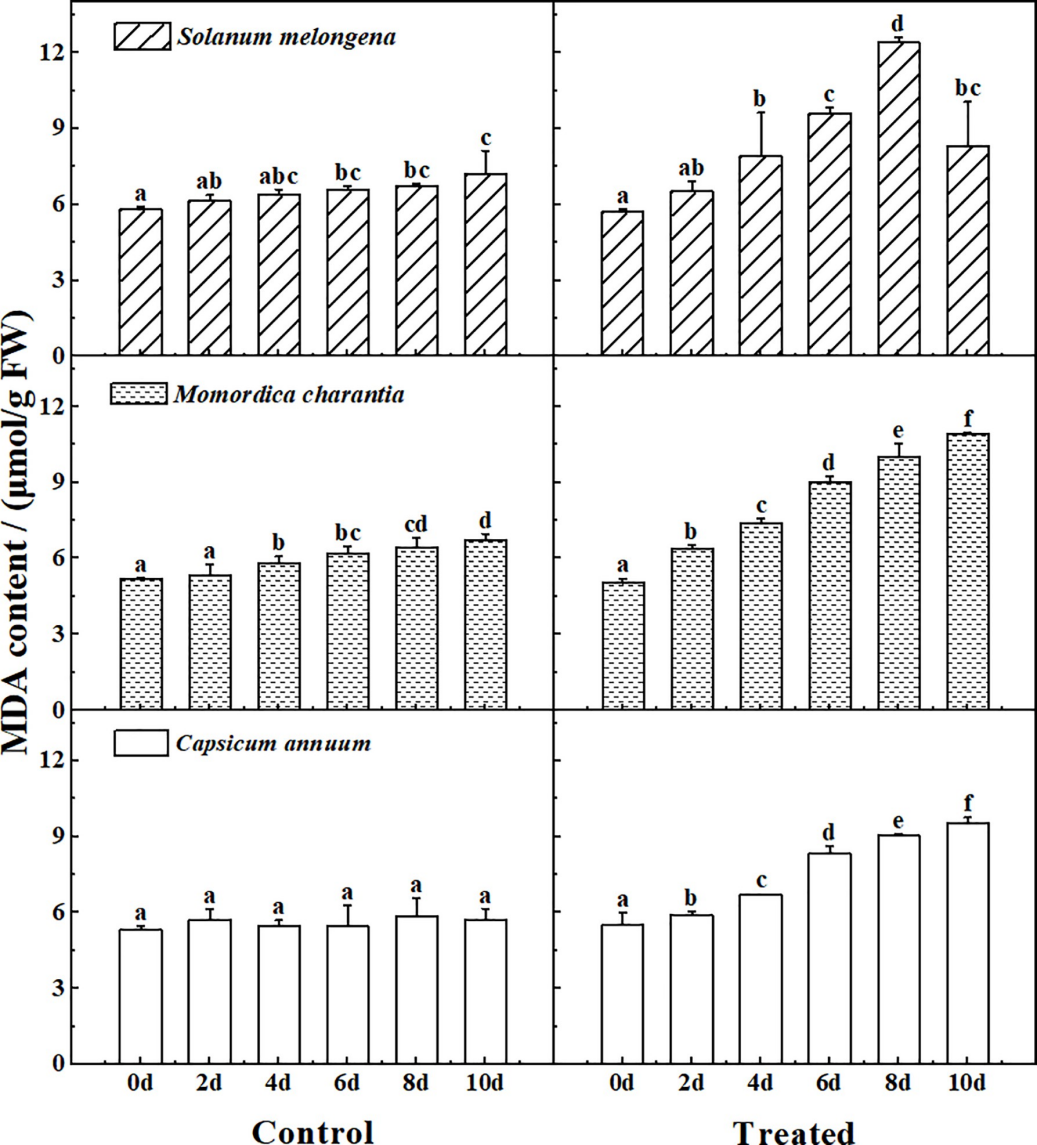
The response of malondialdehyde (MDA) to normal watering (Control) and salt water irrigation (Treated) in *Solanum melongena*, *Momordica charantia*, *Capsicum annuum*. The single salt solution (NaCl, 150 mmol/L) was watered once a day (25 ml per pot) for a total of 10 days, seedling leaves were randomly sampled every 2 days to determine MDA. Error bars represent standard errors of MDA with 4–5 replicates measurements. The same letter in each picture represents no significant difference (Duncan test, P < 0.05, n = 4–5).

Fig 4 shows the MDA determination results of the control and treated group of three cash crops. There was no significant difference in MDA among the three cash crops in the control group. After stress, the MDA content of the three crops all showed a increasing trend gradually, but the content of *Solanum melongena* decreased on the 10th day of stress. Meanwhile, the intraspecific responses of the three plants after treatment were significantly different (P<0.05).

### Soil characteristics

Table 2 shows some soil characteristics (electrical conductivity, pH, salt content) before and after the treatment. After the soil salinity was measured, the soil salinization grade was determined according to the Salinization Grade Standard in Table 1. The background value of salt content in the cultivation soil was 0.97 g/kg in Table 2 (the third row). It shows that there is no salinization in the cultivated soil before salt treatment, and the control group was cultivated under this condition. After 10 days of salt stress treatment for the three cash crops, the soil salt contents are higher than 3 g/kg. Among them, the soil salt content for *Momordica charantia*, *Capsicum annuum* are grade Ⅲ, and for cultivating *Solanum melongena* reached 3.97 g/kg (Salinity Grade Ⅳ). It can be seen from the measurement results that the soil salt content of the control group also decreased after 10 days, but it was not significant. At the end of the stress treatment, the soil salinity of the three cash crops in the treatd group increased sharply, which was significantly different from the control group of various crops (P<0.05). The electrical conductivity of soil also increased significantly (P<0.05). There was no significant change in soil pH. The soil salinization grade has changed greatly (Salinity Grade Ⅰ to Grade Ⅲ or Ⅳ).

**Table 2:** Measurement results of soil characteristics.

Soil Characteristics						
			Electrical Conductivity (μS/cm)	pH	Salt Content (g/kg)	Salinity Grade
Before the treatment			311.33±9.07^a^	7.13±0.06^ab^	0.97±0.05^b^	Ⅰ
After the treatment	*Solanum melongena*	Control	303±6.08^a^	7.1±0.17^a^	0.85±0.01^ab^	Ⅰ
		Treated	855±21.07^c^	7.07±0.06^a^	3.97±0.09^e^	Ⅳ
	*Momordica charantia*	Control	300±12.12^a^	7.33±0.12^b^	0.86±0.02^ab^	Ⅰ
		Treated	906.33±17.21^d^	7.13±0.58^ab^	3.55±0.11^d^	Ⅲ
	*Capsicum annuum*	Control	306.33±7.09^a^	7.1±0.1^a^	0.76±0.03^a^	Ⅰ
		Treated	800.33±11.37^b^	7.1±0.17^a^	3.34±0.14^c^	Ⅲ

*Error bars represent standard errors of electrical conductivity, pH and salt content of soil with 3 replicates measurements. The same letter in each column represents no significant difference (Duncan test, P < 0.05, n = 3).

## Discussion

Chlorophyll is a typical representative indicator of photosynthesis. As the important photosynthetic pigment, it can reflect the photosynthetic energy efficiency. After salt stress, the changes of chlorophyll content of *Solanum melongena* and *Momordica charantia* indicated that they were obviously affected by salt stress. Fewer photosynthesis pigments under salty conditions could reduced the biomass significantly [9, 10]. This decrease may be due to the activity of some chlorophyll degrading enzymes induced by salinity [26–28]. There was no significant difference in chlorophyll content in and between the control and treated groups in *Capsicum annuum*. This shows that *Capsicum annuum* can grow normally in this saline soil (3.34±0.14 g/kg, Salinity Grade Ⅲ) and can maintain the increase of biomass continuously.

Soluble protein and Pro, as osmotic regulators in plants, are important indicators to measure the osmotic adaptability of plants [4, 6–10, 13–15, 17, 19, 21, 22]. They are the important symbol of plant stress response. Plants adjust the content of soluble substances under stress to change the osmotic pressure, maintain the stability and integrity of the cell membrane, ensure the normal physiological activity of enzymes, and maintain the intake and maintenance of water [8, 22, 29, 30]. MDA is the main product of membrane lipid peroxidation, and its content can reflect the degree of damage to plant cells [6, 9]. The higher of the MDA content, the more severely damaged to the cell membrane system.

At the initial stage of stress, the contents of soluble protein, MDA and Pro of the three cash crops all showed an upward trend. It shows that salt stress has negative effects on plants. The accumulation of soluble protein and Pro indicated that the plant had a certain ability to resist salt, and the accumulation of MDA indicated that the cell membrane of the plant was damaged [4, 9, 10, 17, 22]. The change rates of each index in plants were inconsistent. *Solanum melongena* showed the most obvious changes, in which soluble protein increased to 2.1 times, MDA increased to 2.2 times, and Pro increased to 4.2 times. This result indicated that among the three cash crops, *Solanum melongena* was the most sensitive to salt in three corps.

The three physiological indicators respond differently in three plants to salt stress with increasing stress time. Soluble protein of *Solanum melongena* and *Momordica charantia*, Pro of *Momordica charantia* and *Capsicum annuum*, and MDA of *Solanum melongena* decreased in the later stress period.

Pro is widely distributed in plants, its hydration ability is very strong. So Pro plays an important role in resisting environmental stress [8, 15, 21, 31]. The accumulation of Pro can increase the concentration of cell fluid and effectively maintain the osmotic balance. Therefore, when plants are under adversity, such as salt stress, the increase of Pro content may be the performance of plant adaptation or plant cell damage [30–32]. In the later stage of stress, the content of Pro in *Momordica charantia* and *Capsicum annuum* was gradually decreased, but the content values were higher than the control, which may be the gradually adapting to the stress environment in *Momordica charantia* and *Capsicum annuum* [33].

At the same time, the changes of Pro content in the later period of stress may help to explain the change characteristics of soluble protein and MDA in the three crops. The Pro content of *Solanum melongena* increased continuously during the stress process, which stabilized the protein and prevented membrane lipid peroxidation [21]. Therefore, both soluble protein and MDA decreased in the later stage of stress in *Solanum melongena*. However, the changes in the indicators of *Solanum melongena* were relatively large, and were much higher than those of the other two crops, indicating that *Solanum melongena* was extremely sensitive to salt stress. Considering that the chlorophyll of *Solanum melongena* was greatly affected (significantly decreased, P<0.05), we believe *Solanum melongena* has a certain ability to resist salt stress, but cannot maintain normal growth under long-term stress.

In terms of Pro content, there was no significant difference between *Solanum melongena* and *Capsicum annuum*, but the value in the control group was significantly higher than that of *Momordica charantia* (P<0.05). Pro itself can contribute to reactive oxygen species (ROS) scavenging [34], so it indicated that *Solanum melongena* and *Capsicum annuum* itself had better stress resistance than *Momordica charantia*. In the later stage of stress, the decrease of Pro content in *Momordica charantia* and *Capsicum annuum* obviously affected their protective effect on cell membrane, so MDA of both crops continued to rise. At the same time, the decrease of Pro content of *Momordica charantia* affected the stability of the protein [21], so the protein decreased slightly. But for *Capsicum annuum*, although its protective function of Pro is reduced, it still maintains a high protein content, which indicates that various physiological indicators of *Capsicum annuum* work together in the process of stress resistance, and its ability to resist salt stress better than the other two crops.

Then we discuss the desalination capacity of plants. The initial salt content of the soil was the same (0.97g/kg, Salinity Grade Ⅰ). During the 10 days of salt stress, the concentration of the salt solution was the same as the amount of water, so the amount of salt added to each pot was the same value at the end of salt stress. Therefore, the difference in soil salinity measured at the end of the salt stress could reflect the different ability of plants to absorb salt. That is, the ability of plants to remove salt from the soil. Through the comparison of soil salt content after the experiment, it can be seen that the soil salt content of cultivated *Capsicum annuum* is the lowest (3.34±0.14 g/kg, Salinity Grade Ⅲ), and the salt content of *Solanum melongena* is the highest (3.97±0.09 g/kg, Salinity Grade Ⅳ). There is the significant differences among the three crops in soil salinity after treatment (P<0.05). Therefore, it can be considered that the salt removal ability of *Capsicum annuum* is the strongest in three cash crops.

## Conclusions

In this study, the response of three widely grown cash crops to salt stress was studied, and their desalination capacity was analyzed and discussed. Based on the above analysis, the differences in the resistance indicators of the three cash crops can be roughly inferred that their salt tolerance is *Capsicum annuum*> *Momordica charantia*> *Solanum melongena*. *Capsicum annuum* has high salt resistance, which can maintain normal growth under 150 mmol/L salt solution irrigation. Various physiological factors in the plant improve the salt resistance of *Capsicum annuum* through comprehensive action, it can produce more good adaptability with the prolongation of stress time. *Solanum melongena* is more sensitive to salt stress, its growth is greatly affected by salt stress. The results of soil index showed that all three crops had a certain ability to remove salt, and *Capsicum annuum* had the strongest desalination ability. As a high-value cash crop, *Capsicum annuum* has strong salt resistance and good desalination ability. Its economic and ecological benefits are outstanding as a good crop for planting in saline-alkali land.

Under the current situation of increasingly serious soil salinization, the challenge of global food security is increasing. Therefore, it is urgent to improve land utilization and develop crops with strong salt resistance. In addition, according to the NbS concept, using the functions of the ecosystem itself to carry out ecological protection and restoration, plants with high desalination ability can be selected for ecological restoration of saline-alkali land. Combining the above two aspects, it is a very meaningful research direction to develop, select and promote the cash crops with high salt resistance and strong desalination ability.

## Supporting information

S1 Data The data of soil indexes before and after the experiment, and the data of chlorophyll, malondialdehyde, proline, soluble protein physiological indexes of 3 cash crops after the experiment. Mean and standard error (n = 3~5) are shown.
(ZIP)

S2 Data (ZIP)
